# Imputation of low‐density marker chip data in plant breeding: Evaluation of methods based on sugar beet

**DOI:** 10.1002/tpg2.20257

**Published:** 2022-10-18

**Authors:** Tobias Niehoff, Torsten Pook, Mahmood Gholami, Timothy Beissinger

**Affiliations:** ^1^ Animal Breeding and Genomics Wageningen Univ. & Research Postbox 338, 6700AH Wageningen The Netherlands; ^2^ Dep. of Crop Sciences, Division of Plant Breeding Methodology Univ. of Göttingen Göttingen 37075 Germany; ^3^ Dep. of Animal Sciences, Animal Breeding and Genetics Group Univ. of Göttingen Göttingen 37075 Germany; ^4^ RD‐SBCE‐BTA, KWS SAAT SE & Co. KGaA Grimsehlstr. 31 Einbeck 37574 Germany; ^5^ Center for Integrated Breeding Research Univ. of Göttingen Göttingen 37075 Germany

## Abstract

Low‐density genotyping followed by imputation reduces genotyping costs while still providing high‐density marker information. An increased marker density has the potential to improve the outcome of all applications that are based on genomic data. This study investigates techniques for 1k to 20k genomic marker imputation for plant breeding programs with sugar beet (*Beta vulgaris* L. ssp. *vulgaris*) as an example crop, where these are realistic marker numbers for modern breeding applications. The generally accepted ‘gold standard’ for imputation, Beagle 5.1, was compared with the recently developed software AlphaPlantImpute2 which is designed specifically for plant breeding. For Beagle 5.1 and AlphaPlantImpute2, the imputation strategy as well as the imputation parameters were optimized in this study. We found that the imputation accuracy of Beagle could be tremendously improved (0.22 to 0.67) by tuning parameters, mainly by lowering the values for the parameter for the effective population size and increasing the number of iterations performed. Separating the phasing and imputation steps also improved accuracies when optimized parameters were used (0.67 to 0.82). We also found that the imputation accuracy of Beagle decreased when more low‐density lines were included for imputation. AlphaPlantImpute2 produced very high accuracies without optimization (0.89) and was generally less responsive to optimization. Overall, AlphaPlantImpute2 performed relatively better for imputation whereas Beagle was better for phasing. Combining both tools yielded the highest accuracies.

## Core Ideas

- Beagle is sensitive to parameter tuning.
- Best imputation accuracies could be achieved by using a combination of Beagle and AlphaPlantImpute2.
- The population structure influenced imputation accuracy.

**Abbreviations**
API2: AlphaPlantImpute2
DH: double haploid
SNP: single nucleotide polymorphism

## 1 INTRODUCTION

Imputation allows the prediction of missing marker information in data sets. While it is not uncommon for a fraction of markers to have missing calls after genotyping, missing marker information can also be the result of merging two datasets that were genotyped with different marker panels.

Imputation is regularly used as a preprocessing step prior to genetic analyses. For applications that do not allow for missing values, including certain methods for genomic prediction (Meuwissen et al., 2001), imputing genetic raw data generated by single nucleotide polymorphism (SNP)‐chips or DNA sequencing is indispensable. It has also been shown to increase the detection power in genome‐wide association studies (Nyine et al., 2019; Wang et al., 2018; Willer et al., 2008; Yan et al., 2017). Imputation also allows the aforementioned merging of data sets that are both genotyped with different marker panels assuming that a fraction of markers is genotyped in both sets. Geibel et al. (2021) showed how imputation of datasets comprised of sequenced and SNP chip genotyped animals can mitigate ascertainment bias which allows for more accurate estimation of population parameters.

Genotyping allows breeders to estimate genomic breeding values for new individuals at an early stage in a breeding program, thus reducing the generation interval. Basing selection on these estimated breeding values can reduce the amount of resources and time otherwise spent on phenotyping (Windhausen et al., 2012). The accuracy of genomic prediction may increase with the number of individuals in the reference set and the density of markers genotyped albeit the improvement from genotyping additional markers diminishes the more markers are genotyped (Asoro et al., 2011; Erbe et al., 2013; Gorjanc, Battagin, et al., 2017; Hickey et al., 2014; Jacobson et al., 2015; Moghaddar et al., 2015; Norman et al., 2018; Wiggans et al., 2012). The rate of diminishing improvement depends on the population size, structure, and diversity (Gorjanc, Battagin, et al., 2017; Lorenzana & Bernardo, 2009). For maintaining the genomic prediction accuracy across selection cycles, more markers are generally required to better capture the linkage disequilibrium structure (Asoro et al., 2011; Lorenzana & Bernardo, 2009; Norman et al., 2018).

A possible solution to obtain high‐density marker data while spending comparatively little on genotyping is to deliberately genotype only a proportion of the population at high‐density while genotyping the majority at low‐density. The high‐density individuals can then be used as a reference to impute the low‐density individuals to high‐density (Gorjanc, Battagin, et al., 2017; Gorjanc, Dumasy, et al., 2017; Jacobson et al., 2015; Thorn et al., 2021; Wiggans et al., 2012).

Many imputation methods for plant and animal breeding have been developed over the years such as FImpute (Sargolzaei et al., 2014), AlphaPlantImpute (Gonen et al., 2018), AlphaFamImpute (Whalen et al., 2020), AlphaImpute2 (Whalen & Hickey, 2020), OutcrossSeq (Chen et al., 2021), AlphaPlantImpute2 (Thorn et al., 2021), and HBImpute (Pook et al., 2021). The majority of these methods are designed for genotyping only a fraction of the population at high‐density. Often, these are the founders or parents that contribute all or most of the genetic material to the population. Imputation generally works by first detecting haplotypes from a reference set similar to the haplotype of a low‐density individual and then inferring allele states from the similar high‐density haplotype for missing positions on the low‐density haplotype. Offspring individuals can be explained as a mosaic of chromosome segments of a set of founders and/or ancestors. Because the breakage of haplotypes is relatively rare in breeding programs due to the low number of meiosis, parents make a powerful reference set.

Over the years, the software Beagle (Browning et al., 2018) has become a ‘gold standard’ for imputation; new software is typically tested against Beagle as a benchmark for performance (Chen et al., 2021; Korkuć et al., 2019; Sargolzaei et al., 2014; Swarts et al., 2014; Thorn et al., 2021; Wang et al., 2020; Whalen et al., 2018; Whalen et al., 2020; Whalen & Hickey, 2020; Zheng et al., 2018). Beagle was originally developed for use in human genetics (Browning et al., 2018; Das et al., 2018) and its default settings are designed to work well for outbred human populations (Pook et al., 2020). Plant breeding populations, on the other hand, often show much higher levels of inbreeding and lower genetic diversity. Recently, Pook et al. (2020) demonstrated that Beagle imputation can be improved when working with data from breeding populations by tuning the imputation parameters. Thus, benchmarking the performance of Beagle in plant and animal breeding dataset requires adaptation of the parameter settings because the imputation performance in Beagle will otherwise be underestimated. This would lead to a biased comparison between approaches. Only a few studies use imputation parameters other than the default (Arouisse et al., 2020; Geibel et al., 2021; Lamb et al., 2021; Nyine et al., 2019; Thorn et al., 2021; Whalen & Hickey, 2020). These studies lower the parameter for the effective population size and some also increase the window size.

**Core Ideas**

- Beagle is sensitive to parameter tuning.
- Best imputation accuracies could be achieved by using a combination of Beagle and AlphaPlantImpute2.
- The population structure influenced imputation accuracy.

Thorn et al. (2021) recently presented a new method, AlphaPlantImpute2 (API2), and compared it to Beagle version 5.1. In this paper, we investigate the optimization of both tools further and give guidelines to researchers who want to improve their imputation accuracy. We chose API2 because it was developed specifically for agricultural plant breeding data, its ability to work with and without pedigree information and reportedly good performance (Thorn et al., 2021). Beagle was chosen because of its versatility and common use in imputation. To allow for a fair comparison, we first optimized the imputation parameters and procedure for Beagle and API2. We then tested the two tools on simulated populations based on real sugar beet marker data. While API2 performed well with default settings, the accuracy of Beagle 5.1 improved four‐fold when input parameters were optimized, the imputation approach was optimized, and pedigree information was used. We found that higher numbers of low‐density offspring decreased accuracy when imputing with Beagle. As proof and as a solution to counteract this unwanted behavior, we suggest an approach which we call ‘single‐genotype imputation’ which improves accuracies independent of the number of low‐density lines. Furthermore, we observed that Beagle was superior at phasing, whereas API2 was superior at imputation. We used the respective strength of both methods in a combined pipeline we call ‘Beagle+AlphaPlantImpute2’. We show its superiority in terms of run time and accuracy over other methods.

## 2 MATERIALS AND METHODS

Our study can be split into two parts: the optimization of parameters for both imputation tools and the comparison of both tools run with optimized parameters in selected scenarios. Data for both the optimization and the scenario comparison were simulated. To capture the linkage structure as observed in a real breeding population, we used data from real genotyped sugar beet lines as our parents or founders. Our methods are described in detail in the following sections.

### 2.1 Raw data

To simulate the initial data to later compare imputation prediction to, we used proprietary real sugar beet marker data provided by KWS SAAT SE & Co. KGaA. The data set contained bi‐allelic marker information of 1,655 lines, 41 of which are parents, and the remainder are F3 offspring in 67 families. All lines were genotyped with a custom‐made experimental SNP array that contains 19,519 markers covering all nine chromosomes hereinafter referred to as ‘high‐density’ or ‘20k’ panel. The parents showed various degrees of homozygosity based on marker information (76 to 99% with mean of 88%). Together with a genetic map, three low‐density panels containing 950, 1,842 and 2,719 markers were provided hereinafter referred to as ‘low‐density’ or ‘1k/2k/3k’ panel, respectively. The smaller panels are subsets of the larger ones, that is, all markers present on the 1k panel are also present on the 2k and 3k panel.

All lines with more than 10% missing marker information were removed from the original data set. Also, all lines with more than 100 Mendelian errors were removed. For this, the marker states of parents were assumed to be correct. We define a Mendelian error as the presence of an allele that is not present in either of the parents. After all filtering steps, 1,357 lines from 36 families remained in the data set. 11 of the initial 41 parents were removed. About a quarter of all markers were monoallelic but not removed. The average fraction of missing marker information per genotype was 1.4% after all filtering steps.

### 2.2 Simulation of data sets

We phased and imputed the real 1,357 lines with Beagle 5.1 (Browning et al., 2018) in one step with default settings except for the effective population size which was set to 30. After imputation and phasing by Beagle, no missing information was left in the data set.

The phased parental information was then extracted and together with the provided genetic map and pedigree used for simulations with the R package ‘Meiosis’ (Müller & Broman, 2017). No mutations were induced. For traditional population types, the offspring were obtained by simulating the crosses as denoted in the pedigree followed by a number of selfings or double‐haploid induction according to the population type simulated. Unless noted otherwise, five offspring per family were simulated. Biparental intercross populations were created by randomly selecting seeds from the F1 and allowing open‐pollination within the family in the consecutive generations. Biparental intercross populations are denoted as OP10perfamn100 where 10 refers to the number of generations after the F1 and 100 refers to the population size. We also simulated open‐pollinating populations based on all 30 founder lines. For open‐pollinating populations, random mating started already from the parent generation on thus skipping the F1 creation step used for biparental populations. We denote these open‐pollinating populations based on all 30 founders as OP10n100 with 10 referring to the number of generations and 100 to the family size in each generation. An OP10n100 is obtained one generation sooner than an OP10perfamn100 because no designated F1 generation is simulated. These offspring types are simulated based on the phased information of the real genotypes provided by KWS SAAT SE & Co. KGaA. In one scenario, we also investigate the effect of different parent types on imputation. For that, the parents were simulated based on the phased real genotypes too. Consequently, the offspring simulation used the simulated parents. The genotype information of the 1,327 real offspring lines was only used to phase the real parent lines. More details about this scenario can be found in Section 2.6. Table 1 provides an overview of the populations used in this study.

**TABLE 1:** Overview of populations used. The generations refer to the number of generations separating the population from the generation it is based on

Offspring type	Based on	Mode of pollination	Generations	Population size	No. of families	Population type
F1	parents	cross	1	5 per family	36	traditional
F2	biparental F1	selfing	1	5 per family	36	traditional
F3	biparental F1	selfing	2	5 per family	36	traditional
F10	biparental F1	selfing	9	5 per family	36	traditional
DH	biparental F1	/	1	5 per family	36	traditional
OP10perfamn5	biparental F1	open‐pollination within family	10	5	36	biparental intercross
OP10perfamn10	biparental F1	open‐pollination within family	10	10	36	biparental intercross
OP10perfamn100	biparental F1	open‐pollination within family	10	100	36	bi‐parental intercross
OP100perfamn100	biparental F1	open‐pollination within family	100	100	36	bi‐parental intercross
OP10n5	all 30 parents	open‐pollination	10	5	1	open‐pollinating
OP10n10	all 30 parents	open‐pollination	10	10	1	open‐pollinating
OP10n100	all 30 parents	open‐pollination	10	100	1	open‐pollinating
OP100n100	all 30 parents	open‐pollination	100	100	1	open‐pollinating

For comparability, we kept the number of lines constant for all population types, that is, we increased or decreased the total number of offspring lines to 180 in the last generation of open‐pollination.

Simulating datasets with different homozygosity levels for the parents was done by simulating open‐pollination between all 30 parents thus also allowing selfing. For the female side, we implemented restrictions of contributions to the next generation by mimicking female gametic control to reduce the change of allele frequencies due to drift. For female gametic control, one seed per parent line was collected to replace the original parent. These seeds were considered F1's. Different parent population types like F2 or double haploids (DH) were created the same way as explained for the offspring. Simulated parents are thus also based on the real parental lines. The simulation of parents was done to investigate the effect of homozygosity in parents. Basing these simulations on the phased data of real lines has the advantage of capturing the low‐density structure of a real breeding population. Whenever the original parents provided by KWS SAAT SE & Co. KGaA were used in this study, they are denoted as ‘real.’

For simplicity, this study uses the term ‘line’ to refer to a genotypically distinct plant genotype regardless of its heterozygosity level. The parents of a line in this study are defined as those lines to whom cross the line in question can be traced back. The line in question may be the result of the combination of the gamete of its parents (F1) or may be separated from the cross by any number of meiotic events like selfing.

### 2.3 Data set modification

Genotyping at low‐density was mimicked by masking all markers not present on the low‐density panel. The data of designated low‐density lines was masked.

In a scenario investigating the influence of missing genotype calls, the markers at which to remove information were sampled randomly per line. The fraction of missing markers was kept identical for all lines. In the scenario testing the influence of incorrect marker calls, we randomly sampled the markers per line at which the genotype state was to be changed. For every marker that was to be changed, we randomly set the genotype state to 0, 1 or 2 and never left the marker state as the true value. For example, if the true state is 0, only 1 or 2 were candidates. The fraction of errors induced prior to masking was identical for all lines.

Every combination of factor levels was tested with five replicates. A new data set was simulated for every replicate. The data sets were simulated to mimic the following type for parameter optimization and procedure optimization. It was also used for the scenarios tested later with modifications described in Section 2.6. This population type corresponds to row 3 in Table 1:

‐ 30 real parents genotyped at high‐density (not simulated)
‐ 36 families with five offspring per family
‐ F3 offspring genotyped with the 1k SNP panel
‐ No error induction
‐ No missing genotype call induction.

### 2.4 Parameter optimization

The type of data set used to optimize parameters is our standard data set type described in Section 2.3.

**FIGURE 1:**
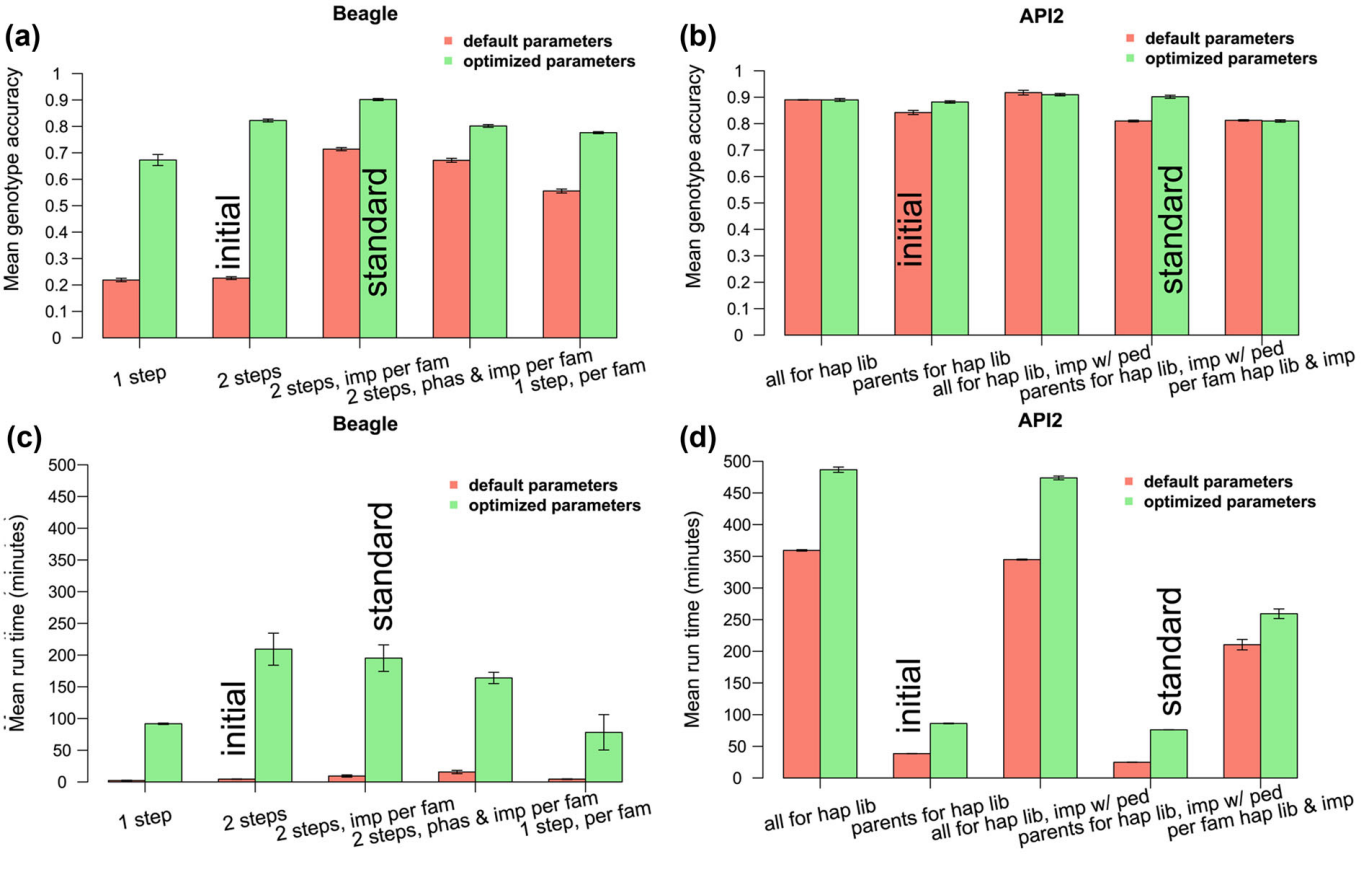
Accuracies (a, b) and run times (c, d) observed for imputation with default and optimized parameters as shown in Table 2 and Table 3 for different imputation procedures with AlphaPlantImpute2 (b, d) and Beagle (a, c; each with replicates). ‘initial’ marks the procedure used as a starting point for optimization. ‘standard’ marks the procedures used for all following tests unless mentioned otherwise. ‘2 steps’ refers to first running Beagle for phasing and then using the phased high‐density parents as reference for imputation in a second step. ‘All’ and ‘parents’ for AlphaPlantImpute2 state what genotypes were considered for the creation of the haplotype library. ‘hap lib’ = haplotype library, ‘imp’ = imputation, ‘phas’ = phasing, ‘per fam’ = phasing or imputation restricted to only the genotypes and two parents of a family, ‘w/ ped’ = with pedigree and/or founders information. Error bars indicate standard error of the mean

To allow for a fair comparison between API2 (Thorn et al., 2021) and Beagle 5.1 (beagle.18May20.d20), we first optimized the parameters for both tools. For API2, this was done with a procedure in which only the unphased high‐density parent lines of the simulated offspring lines were used to create the haplotype library by setting ‘hd_threshold’ to 0.8. This library was then used for low‐density offspring imputation without pedigree information. For parameter optimization in Beagle, we used a two‐step method in which all high‐density and low‐density data was imputed and phased by Beagle once. Then, only the phased information of the high‐density lines was extracted and given as reference to a second Beagle run in which the low‐density unphased data was imputed. The imputation schemes labelled as ‘initial’ in Figure 1 are the ones described above.

Parameters were optimized iteratively based on an incomplete grid search. The parameters were always set to be identical for the haplotype library creation step (API2) or phasing of parents (Beagle) and the imputation step. To derive parameters that are not different to default by chance, potentially better parameters were validated in all combinations with default parameters, except for the parameters ‘ne’ and ‘window’ of Beagle, as modifying these had been found to already have a large positive effect on accuracy (Pook et al., 2020).

### 2.5 Imputation procedures

After the optimized parameters were found, we tested different imputation procedures, or pipelines. The imputation procedures and pipelines were never provided with the true phase states.

To mimic the use of pedigree information in Beagle, a reduced reference panel that only contained the relevant parents was used, that is, the parents of a family were looked up externally in R. Then, the reference set was restricted to only the parental lines of the family while the set that was to be imputed was restricted to only the offspring lines. This was repeated for all 36 families. We call this procedure ‘familywise imputation’. To check if imputation results can be improved further, we also did the phasing step familywise in addition to the familywise imputation step. To check whether running Beagle in two steps is really necessary, we also tried familywise imputation without the phasing step, that is, no reference provided.

In API2, we tested a procedure in which all high‐density and low‐density lines are used for the haplotype library creation step by the software followed by imputation with pedigree information by specifying the founders file. We then tested a procedure in which only the parental lines were used for the haplotype library creation. In yet another procedure, to utilize pedigree information in the haplotype library creation step, we restricted lines specified for library creation to only lines of a family including both low‐density and high‐density lines. Imputation was then also done per family and the process repeated for all families. This is comparable to the two‐step approach in Beagle where phasing and imputation are done familywise.

Based on our findings, we developed the single‐genotype imputation pipeline for Beagle. Single‐genotype imputation with Beagle is like the two‐step procedure without a pedigree as used for optimizing parameters, but instead of specifying all low‐density lines in the second step, only one line is specified at a time. The procedure was repeated for all low‐density lines. For the creation of the reference set, only high‐density lines were considered. For single‐genotype imputation, optimized parameters were used except for ‘iterations,’ which was found to be sufficient when left at default.

The pipeline Beagle+API2 aims at combining the strengths of both tools that we have identified in scenarios tested before and are presented chronologically in this study. In this pipeline, only high‐density lines are provided to Beagle in a first step. After they are phased, the lines are used as the haplotype library in API2 for imputation of low‐density lines. No pedigree information was utilized in either step. For Beagle+API2, the optimized parameters were used except for ‘iterations’ in Beagle which was found to be sufficient when left at default.

### 2.6 Scenarios

After parameter optimization, the following scenarios were tested with Beagle 5.1 and API2:

1. Marker density on the low‐density panel with the 1k, 2k and 3k panel;
2. Various numbers (0, 1, 2, 3, 4) of the five offspring lines per family genotyped at high‐density;
3. Various fractions (0, 0.001, 0.005, 0.01, 0.02, 0.035, 0.05, 0.1) of missing genotype calls per line;
4. Various fractions (0, 0.001, 0.005, 0.01, 0.02, 0.035, 0.05, 0.1) of genotyping errors per line;
5. Different population types (as labelled in Table 1) with every combination of offspring types with parent types offspring types: F1, F2, F3, F10, DH (traditional) OP10perfamn5, OP10perfamn10, OP10perfamn100, OP100perfamn100 (bi‐parental intercross) OP10n5, OP10n10, OP10n100, OP100n100 (open‐pollinating) parent types: real, F1, F2, F3, F10, DH;
6. Imputing low‐density parent genotypes based on five offspring per family with various numbers of offspring lines per family genotyped at high‐density (1, 2, 3, 4, 5); and
7. Varying numbers of low‐density offspring lines per family (2, 3, 4, 5, 7, 10, 15, 20, 25, 30, 35, 50).
8. Imputing five low‐density F3 offspring lines per family (36 families) based on all 30 real high‐density parent lines;
9. Imputing 50 low‐density F3 offspring lines per family (36 families) based on all 30 real high‐density parent lines;
10. Imputing five low‐density DH offspring lines per family (36 families) based on all 30 F1 high‐density parent lines;
11. Imputing five low‐density F1 offspring lines per family (36 families) based on all 30 DH high‐density parent lines; and
12. Imputing all 30 real low‐density parent lines based on one high‐density F3 offspring line per family (36 families).

For scenarios 1–5 and 7, imputation was done familywise with the two‐step procedure in Beagle. The procedure used with API2 in these scenarios, used only high‐density lines for haplotype library creation and the founder file was provided for imputation. In Scenario 6, pedigree information was not used. Thus, the two‐step procedure without familywise imputation was used for Beagle. The founders file was not specified to API2 for Scenario 6. The optimized parameters were used in these scenarios.

The pipelines for single‐genotype imputation with Beagle and Beagle+API2 were only tested on a few selected scenarios that have previously been found to be challenging:

AlphaPlantImpute2 and Beagle were also tested on these scenarios (8–12). For API2, the procedure considering only high‐density lines in the haplotype library creation step was used. For Beagle, the two‐step procedure was used with the modification that the low‐density lines were not used in the first step which is creating the reference set. In all scenarios, all pipelines were run without pedigree information.

The standard dataset type as described in Section 2.3 was simulated and only the aspects described in the scenarios were modified.

### 2.7 Beagle versions

Two later versions of Beagle are available (5.2 and 5.3). We tried optimizing parameters for these two versions as well to not miss out on recent developments. The two‐step procedure as used in Beagle 5.1 before was used for version 5.2 and 5.3 too. Beagle version 5.2 (beagle.28Jun21.220), as presented by Browning et al. (2021), and Beagle version 5.3 (beagle.08Feb22.fa4) were used. For version 5.3, we always set the parameter ‘em’ to ‘false.’

### 2.8 Implementation

Custom R scripts were written for the imputation procedures, data handling and analysis. The imputation procedure to use, parameters, and data set style were specified in custom made excel tables as input for the R scripts. R version 4.0.2 (R Core Team, 2020) was used. Computation was performed on the high‐performance computing cluster of the Gesellschaft für wissenschaftliche Datenverarbeitung mbH Göttingen using Scientific Linux 7.9. The scripts and input tables for the imputation pipelines are available at https://github.com/tobiasniehoff/imputation_plant_breeding.

All imputation runs were assigned 8 GB of memory and four cores Broadwell Intel E5‐2650 v4 with 2.2‐GHz base frequency.

### 2.9 Performance measurement

The accuracy of imputation was measured as the Pearson correlation per line between the true simulated genotype states and the imputed genotype states. All markers on the high‐density panel were considered. The reported figure in this paper reflects the average accuracy over all low‐density lines. The run time was calculated as the difference of elapsed wall clock time between end and start of running the software, that is, excluding all outside handling.

## 3 RESULTS

### 3.1 Optimizing imputation

#### 3.1.1 AlphaPlantImpute2

Imputation accuracy achieved by API2 with default parameters was 0.89 when all lines were provided for haplotype library creation (Figure 1b). This is one of the highest accuracies of all tested procedures in Beagle and API2, even when no pedigree information is supplied. The run time for default parameters with this procedure was about 360 min. Excluding low‐density offspring from the reference library creation step, i.e., only using the parents, drastically reduced the run time to 39 min while achieving an accuracy of 0.84 (Figure 1b, 1d). We optimized parameters for this procedure. The optimized parameters used for API2 imputation are presented in Table 2. With optimized parameters, this procedure yielded an accuracy and run time of 0.88 and 86 min, respectively. Supplying pedigree information and using high‐density and low‐density lines for library creation yielded accuracies of 0.92 and 0.91 and run times of 345 and 374 min for default and optimized parameters, respectively. When supplying pedigree information, reducing the lines for the library creation step to only high‐density lines lowered the accuracy for default parameters to 0.81 but optimized parameters maintained the accuracy at 0.9 (run times are 25 and 76 min, respectively; Figure 1b, 1d). This procedure with optimized parameters is used as the standard in this study (marked with ‘standard’ Figure 1b, 1d). When the library creation step and the imputation step are carried out per family, the accuracies for default and optimized parameters were both 0.81 while the run times were 210 and 260 min, respectively.

**TABLE 2:** Parameters considered for finding optimized parameters for AlphaPlantImpute2. To only use high‐density lines for the creation of the haplotype library, the parameter ‘hd_threshold’ was set to 0.8

Parameter	Default	Range tested	Optimized	Brief description^a^
n_haplotypes	100	10–500	50	number of similar haplotypes to sample from library for a given window of focal haplotype
n_sample_rounds	10	2–70	50	number of iterations for library refinement
n_windows	5	2–20	default	number of windows a chromosome is divided into
error	0.01	0–0.1	default	genotyping error rate
recomb	1	0.1–2	default	recombination rate per chromosome

^a^ The parameter description is taken from the handbook of AlphaPlantImpute2 (https://github.com/AlphaGenes/AlphaPlantImpute2).

#### 3.1.2 Beagle

Compared with 1 step, running Beagle 5.1 in two steps to separate the phasing of parents from imputation of progeny did not change accuracies much when default parameters were used (0.22 for one‐step vs 0.23 for two‐step with run times of 2.3 and 4.5 min, respectively). With parameters optimized for the two‐step procedure, accuracies obtained with the one‐step procedure increased to 0.67 and 0.82 for the two‐step procedure with respective run times of 92 and 209 min. By doing the imputation step familywise in the two‐step procedure, that is, restricting the set to be imputed to low‐density sibling lines and restricting the reference to the two respective parents, the accuracy could be further increased to 0.9 for optimized parameters and 0.71 for default parameters with run times of 9.3 and 195 min, respectively. This procedure with optimized parameters is used as the standard in this study (marked with ‘standard’ Figure 1a. 1c). Doing the phasing and imputation step familywise yielded accuracies of 0.67 and 0.8 for default and optimized parameters, respectively. This procedure is considering the same information at every step as the API2 procedure labeled ‘per fam hap lib & imp’.

Running Beagle in only one step familywise (rightmost pair of bars in Figure 1a. 1c) resulted in accuracies of 0.55 and 0.78 for default and optimized parameters, respectively.

The optimized parameters for Beagle are presented in Table 3.

**TABLE 3:** Values considered for finding optimal parameters in Beagle 5.1

Parameter	Default	Range tested	Optimized	Brief explanation^a^
ne	1,000,000	4–1,000,000	4	effective population size
window	40	1–400	100	length in cM of each sliding window
burnin	6	1–1,000	90	number of burnin iterations to estimate initial haplotype frequency model for inferring genotype phase
iterations	12	2–20,000	5,000	number of iterations to estimate genotype phase
phase‐states	280	2–10,000	30	number of model states used to estimate genotype phase
imp‐states	1,600	2–5,000	default	number of model states used to impute ungenotyped markers
imp‐segment	6	0.1–700	default	minimum cM length of haplotype segments that will be incorporated in the HMM state space for a target haplotype
imp‐step	0.1	0.001–3	default	length in cM of the step used for detecting short IBS segments
imp‐nsteps	7	1–4,000	default	number of consecutive steps (see imp‐step argument) that will be considered when detecting long IBS segments
cluster	0.005	0.00001–10	default	maximum cM distance between individual markers that are combined into an aggregate marker when imputing ungenotyped markers
overlap	4	0.01–20	default	cM length of overlap between adjacent sliding windows

*Note*. Parameters not shown were not tested and their default values were used. The parameters were always identical for the phasing and the imputation step in the two‐step procedure. Imputation was not done familywise for optimizing parameters.

^a^ The parameter description is taken from the handbook for Beagle 5.1 (https://faculty.washington.edu/browning/beagle/b5_1.html).

Note that although low‐density genotypes were used for the creation of a phased reference set with Beagle, they were removed manually prior to supplying the reference set to Beagle in the second step, that is, low‐density lines were not provided as reference. The same was the case for API2 whenever low‐density genotypes are used in the haplotype library creation step.

**FIGURE 2:**
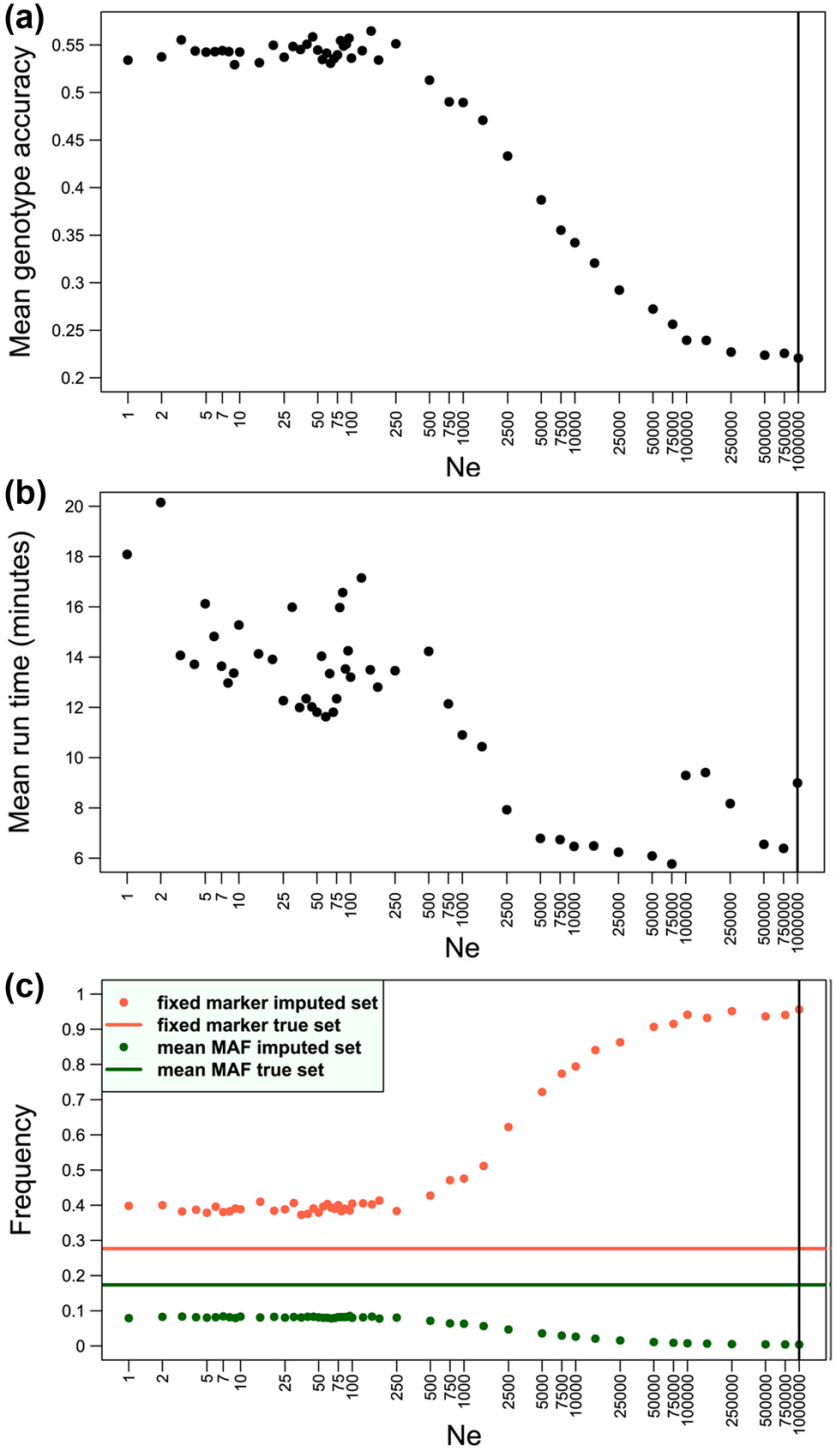
Effect of different values for ‘ne’ on imputation accuracy (a), run time (b), and average minor allele frequency and frequency of fixed loci (c) when imputing with Beagle. All other parameters are set as default. The imputation procedure used Beagle 5.1 in two steps without utilizing pedigree information (noted as ‘2 steps’ in Figure 1a). Black line in (a) and (b) indicates default value (1,000,000). (c) shows the mean minor allele frequency (MAF) and frequency of fixed markers in true and imputed data sets. Mean MAF and frequency of fixed (invariable) markers of true sets are shown as mean values over all sets for display purposes as the variance of these figures due to random mendelian sampling in F3 simulation is negligible

We found that varying the parameter for the effective population size (‘ne’) had a strong effect on imputation accuracy with smaller values increasing the accuracy. No trend could be found for the population used by us for ne values in the range of 1 to 250 (Figure 2a). However, the run time increased when decreasing values for the effective population size were used (Figure 2b).

Another Beagle parameter with a high impact on imputation accuracy is ‘iterations.’ Increasing the number of iterations resulted in improved accuracies. For the data sets used in this study, no further increasing trend could be observed after about 3,000 iterations (Supplemental Figure S1a). The run time increased linearly the more iterations were used from about 8 min for 12 iterations (default) to about 690 min for 10,000 iterations (Supplemental Figure S1b).

The minor allele frequency (MAF) and the frequency of markers that are fixed were calculated in all tests conducted including the scenarios presented later in this study. Regardless of the software, the scenario and the imputation procedure or parameters used, the average MAF over all markers was always lower in the imputed set than in the simulated (true) set. The frequency of markers which are fixed, that is, are invariable in the whole population (excluding high‐density genotypes), was always found to be higher in the imputed set than in the true data set. With higher accuracies, both the mean MAF and the frequency of fixed markers converge towards the true value. These trends are visualized in Figure 2c for the example of decreasing ‘ne’ in Beagle.

### 3.2 Effect of marker density and genotyping offspring at high‐density

**FIGURE 3:**
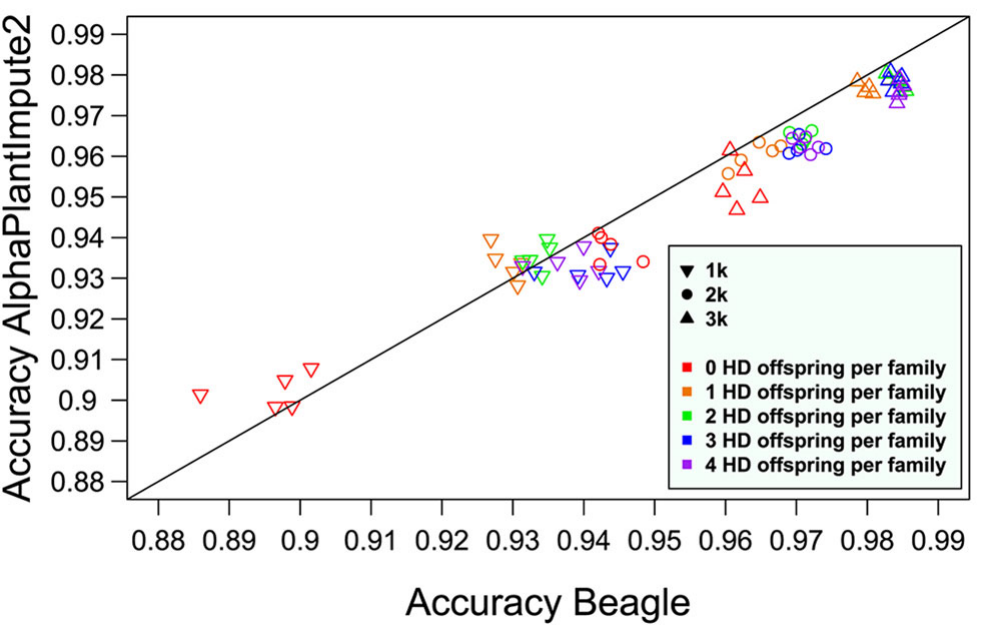
Effect of varying numbers of marker on the low‐density panel and genotyping offspring at high‐density (HD). Always five offspring per family were simulated of which some were genotyped at high‐density. The accuracy is based on only low‐density offspring

Accuracies achieved with Beagle and API2 could both be improved by including more markers on the low‐density marker array (see Figure 3). Additionally, genotyping some offspring at high‐density improved imputation accuracies. However, the additional gain in accuracy decreased the more offspring were already genotyped at high‐density. AlphaPlantImpute2 was superior for the smallest low‐density panel and the lowest number offspring high‐density offspring whereas Beagle was relatively better for higher‐densities and more high‐density offspring lines (Figure 3).

Note that one high‐density line per family means 36 high‐density lines at the population level which can be used in the phasing step in the Beagle procedure. For API2, the high‐density offspring were included in the creation of the haplotype library in addition to the parents. For this scenario, the high‐density offspring were included in the references of the families they were part of. Because the founders were specified for API2, effectively only the two respective parents are used from the haplotype library for imputation whereas Beagle could sample haplotypes from all high‐density family members.

The run time of API2 increased by about 80 min for every additional line per family genotyped at high‐density. No trend in change of run time was observed for imputation with Beagle.

### 3.3 Missing genotype calls and genotyping errors

**FIGURE 4:**
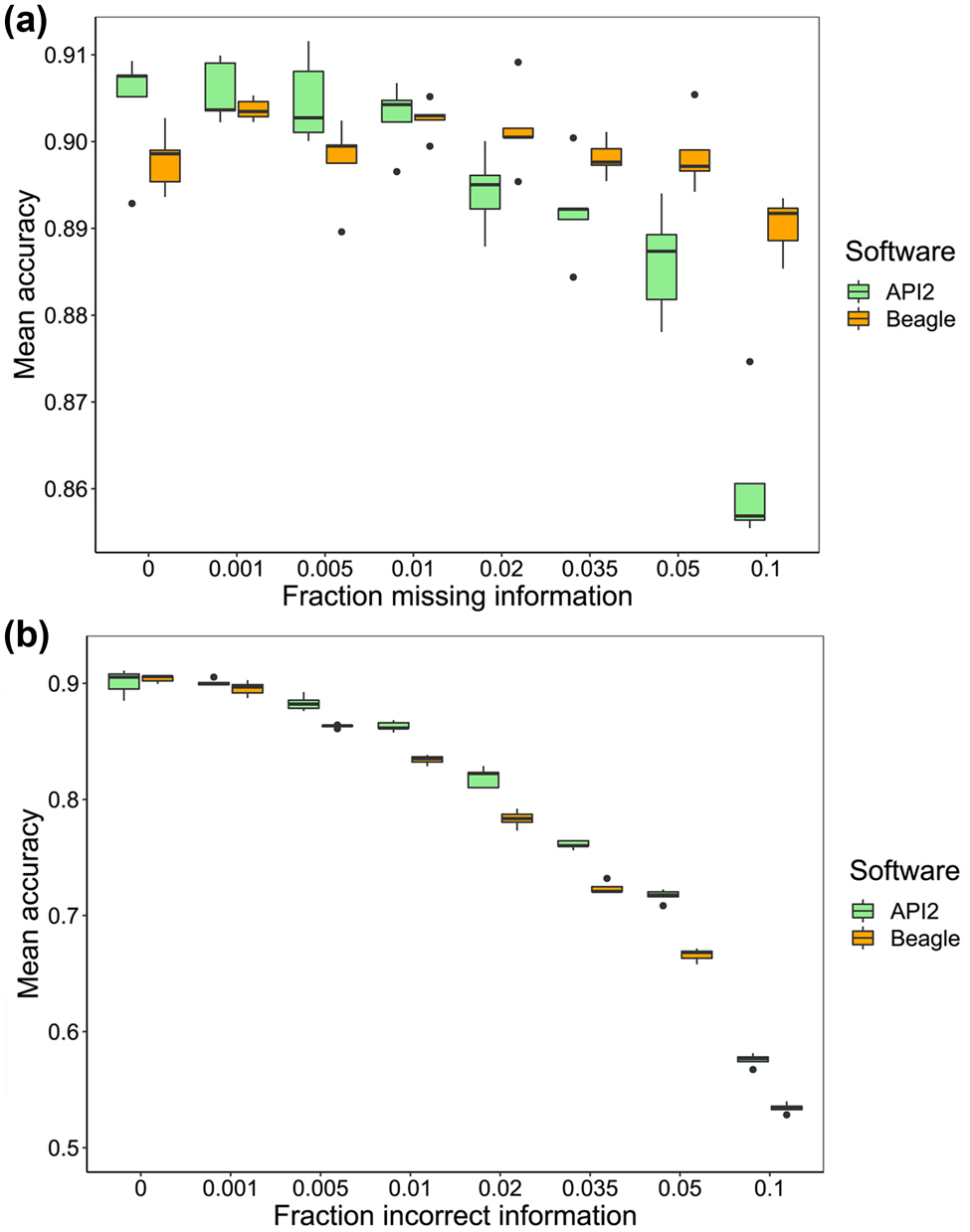
Effect of the fraction of sporadically missing genotype calls in low‐ and high‐density lines (a), and effect of the fraction of random genotyping errors on imputation accuracy (b)

Both, API2 and Beagle were able to make accurate imputation predictions when randomly placed missing genotype calls were induced in the data set for fractions of missingness lower than 2% (Figure 4a). At a missing rate of 0.1, the Beagle procedure achieved an average accuracy of 0.89 whereas API2 yielded 0.86.

With randomly placed incorrect marker information, the accuracies for both software decreased more than for comparable fractions of missingness (compare Figures 4a and 4b). At 0% genotyping errors, accuracy for each software was 0.9 and with 10% genotyping errors, the accuracies were 0.53 (Beagle) and 0.58 (API2).

### 3.4 Population type

**FIGURE 5:**
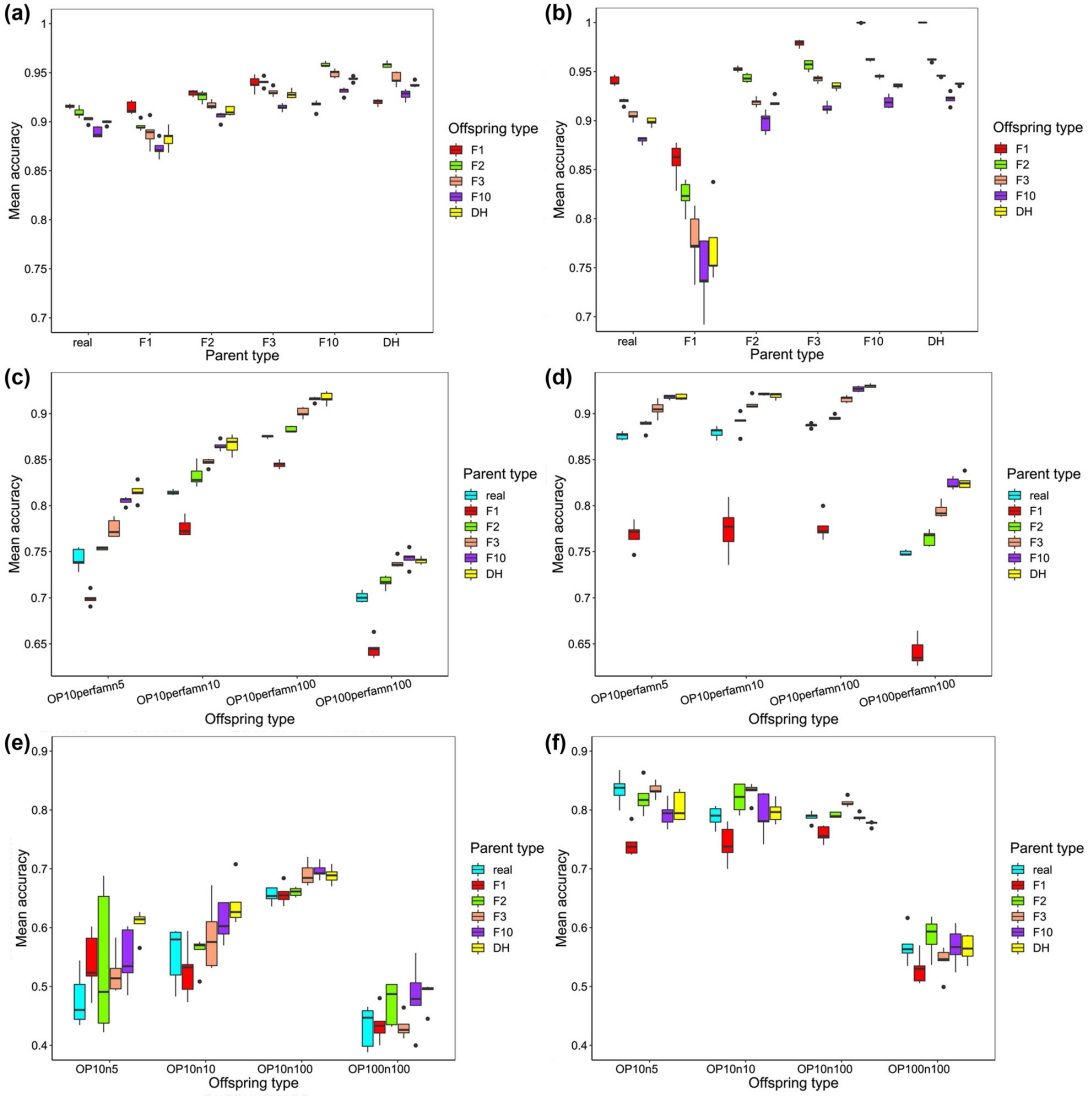
Effect of different population types of parents and offspring on mean accuracy. (a), (c), and (e) refer to imputation results with Beagle; (b), (d), and (f) refer to imputation with AlphaPlantImpute2 (API2). DH, double haploid

Figure 5 shows the accuracies for different parent and offspring type combinations. For biparental populations, imputation was done either familywise (Beagle), or the parents were specified for each line with a founders file (API2). For open‐pollinating populations based on 30 founders like OP10n5(10 generations of open pollination at population size five), considering the pedigree was not possible, that is, the procedures used were those marked as ‘initial’ in Figure 1 together with optimized parameters. For multi‐parent populations, 180 offspring were always imputed regardless of the population size simulated during open‐pollination generations.

The imputation accuracies for Beagle and API2 increased with higher levels of homozygosity in parents (Figure 5). For offspring, the opposite trend was observed. DH offspring resulted in higher accuracies than F10 offspring. When using Beagle, the accuracies for F1 progeny was the highest for the parent types real, F1, F2, and F3 but were the lowest for the highly homozygous types F10 and DH (Figure 5a).

For open‐pollinating and biparental intercross populations, the accuracies were better the fewer generations separated the focal population from the parents (compare OP10perfamn100 with OP100perfamn100 in Figure 5c and 5d and OP10n100 with OP100n100 Figure 5e and 5f). A positive relationship between historic population size and imputation accuracy was observed for Beagle but not for AlphaPlantImpute2.

Generally, accuracies of API2 exceeded or were comparable to the ones obtained with Beagle with the exception of populations based on F1 parents. AlphaPlantImpute2 was able to perfectly impute F1 offspring derived from DH parents whereas Beagle achieved only a mean accuracy of 0.92 (compare Figures 5a and 5b). Populations with DH offspring based on F1 parents are one of the traditional population types that yielded one of the lowest accuracies both with Beagle and API2 (0.88 and 0.77, respectively; Figure 5a and 5b).

For further investigation, the procedures were changed so that the low‐density lines were not included in the population specified to Beagle for phasing. Low‐density lines were not used for the creation of the haplotype library by API2 according to the standard procedure. For investigation, we changed the API2 procedure so that low‐density lines were utilized as well. Note that even though low‐density lines were used for the creation of a haplotype library (API2) or phased reference set (Beagle), they were removed from those reference sets for the second step of imputation in the procedures of both software.

Excluding low‐density lines from the phasing step of Beagle increased accuracies to about 0.91 for populations of DH offspring derived from F1 parents. Including low‐density lines in the haplotype library creation step of API2 increased the accuracy to 0.85 (see Supplemental Figure S2). The accuracy of API2 improved, however, at the expense of run time which increased from 76 to 475 min.

### 3.5 Reconstruction of parents

**FIGURE 6:**
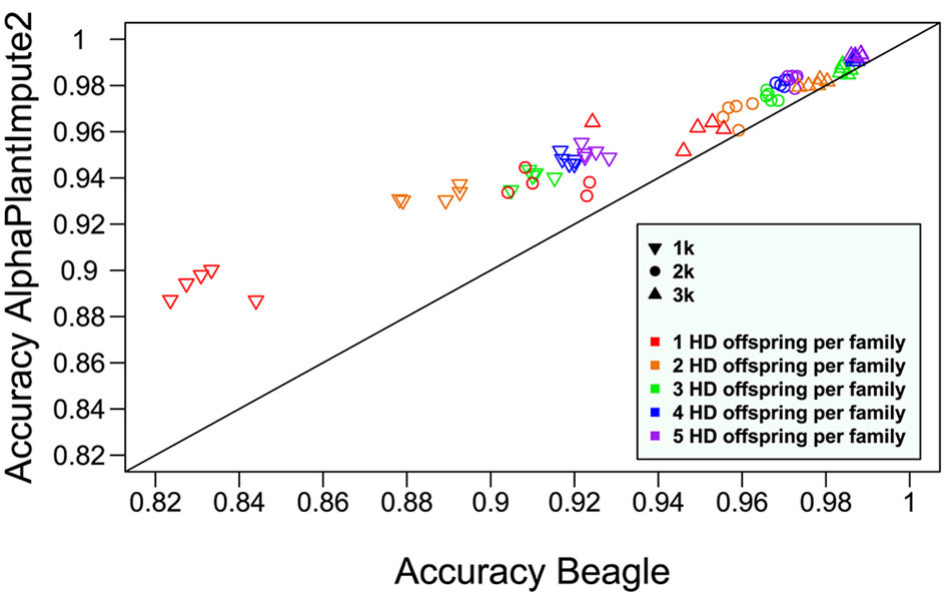
Effect of number of high‐density genotyped offspring (1 to 5) and low‐density panel (1k, 2k, 3k) on imputation accuracy when imputing low‐density parents. Each family has five offspring lines of which some are genotyped at high‐density. Accuracy is based on imputed parents only. Imputation was not done familywise. API2, AlphaPlantImpute2

Figure 6 shows the ability of AlphaPlantImpute2 and Beagle to impute low‐density parents. Accuracies achieved with API2 are better than those achieved with Beagle for all factor level combinations. The relative advantage of AlphaPlantImpute2 was highest for the 1k low‐density panel and 1 offspring per family genotyped at high‐density with an accuracy of 0.89 versus 0.83 for Beagle.

The run time of API2 increased by about 80 min for every additional line per family genotyped at high‐density starting at 100 min. When imputing with Beagle, a decrease in run time could be observed the more lines are genotyped at high‐density with 270 min for one high‐density line per family and 180 min for all offspring genotyped at high‐density. No effect of the marker number of the low‐density panel on run time was found for API2 and Beagle.

### 3.6 Family size

**FIGURE 7:**
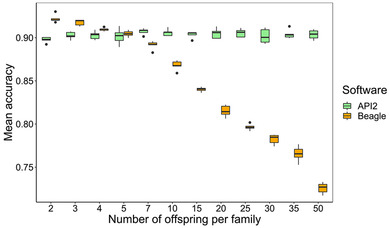
Effect of number of offspring per family on imputation accuracy. API2, AlphaPlantImpute2

For all tested family sizes, the accuracies achieved when imputing with API2 remained constant at 0.9. For Beagle, on the other hand, the imputation quality decreased the more offspring are included in the imputed dataset (Figure 7). For Beagle, imputing data sets with five offspring per family (210 lines in total) took about 300 min whereas the run time increased to about 1,740 min when families consisted of 50 offspring (1,830 genotypes in total). For API2, the run time increased by only about 5 min, from 75 to 80 min.

The procedure presented here for Beagle imputation restricts the reference set to the two parents of a family. Another difference compared with non‐familywise imputation (procedure ‘2 steps’ in Figure 1a) is that the genotype set to be imputed in the second step contains fewer lines because it is restricted to a family. To test whether the restriction to the two parents or the size reduction of the genotype set is responsible for the higher accuracy in our familywise imputation method compared with non‐familywise imputation, we tested familywise imputation with all 30 parents in the reference. Thus, this procedure has the same size of the genotype set as our standard familywise procedure (i.e., 5) but the reference is identical to non‐familywise imputation. The accuracies of this altered procedure were almost as high as with familywise imputation (Supplemental Figure S3). This suggest that the reduction of the reference set to the ancestors only has a minor effect in the familywise imputation procedure.

### 3.7 Pedigree‐free tests and new methods

The Beagle procedure used in this section is the one labeled ‘2 steps’ in Figure 1 with the modification that low‐density offspring genotypes are excluded from the creation of the reference set. The latter has been found to result in equal or better accuracies than if low‐density genotypes were used for the reference set creation (Supplemental Figure S2). The API2 procedure used is the one labeled ‘parents for hap lib’ in Figure 1. Thus, Beagle and API2 considered exactly the same information at every step. The optimized parameter settings were used.

We also tested two new pipelines, single‐genotype imputation with Beagle 5.1 (‘single genotype imputation’), that is, only one line is imputed at a time, and Beagle 5.1 combined with API2 (‘Beagle+API2’), that is, Beagle is used for phasing and API2 for imputation. For these pipelines, the ‘iterations’ parameter of Beagle was changed to 12 (default). All other parameters are identical to the optimized ones presented in Tables 2 and 3.

Table 4 shows the accuracies achieved for all tested methods. The best accuracies for all scenarios were obtained with the Beagle+API2 method. Single‐genotype imputation outperformed the two‐step procedure of Beagle. Not only was the Beagle+API2 method best in terms of accuracy, but it was also the fastest (Supplemental Table S1). To impute five F3 offspring per family (36 families) based on real parents, Beagle+API2 needed about 14 min followed by API2, Beagle, and single‐genotype imputation with Beagle which took 87, 187, and 204 min, respectively.

**TABLE 4:** Mean genotype accuracies for different imputation methods testes in different scenarios

Parents	Offspring	No. of offspring per family	API2	Beagle	Single genotype imputation Beagle	Beagle (phasing) + API2 (imputation)
real (HD)	F3 (LD)	5	0.88 (0.0013)	0.82 (0.0031)	0.87 (0.0018)	**0.89** (0.0006)
real (HD)	F3 (LD)	50	0.88 (0.001)	0.67 (0.0059)	0.87 (0.0006)	**0.89** (0.0008)
F1 (HD)	DH (LD)	5	0.77 (0.137)	0.85 (0.0035)	**0.88** (0.0032)	**0.88** (0.0018)
DH (HD)	F1 (LD)	5	0.91 (0.0021)	0.79 (0.0041)	0.9 (0.005)	**0.98** (0.0011)
real (LD)	F3 (HD)	1	**0.9** (0.0032)	0.87 (0.0038)	0.86 (0.0044)	**0.9** (0.0043)

*Note*. Standard error of the mean shown in parentheses. Best accuracies per scenario are in bold. HD, high‐density; LD, low‐density; API2, AlphaPlantImpute2.

AlphaPlantImpute2 performed worse than Beagle+API2 when imputing F1 lines based on DH parents (0.91 vs 0.98). Manual inspection revealed that in this scenario, API2 sets one chromosome per pair to missing in the haplotype library for the first three chromosome pairs. The other chromosome is represented correctly and chromosomes 4 to 9 are represented in the haplotype library without any missing states.

### 3.8 Beagle versions

We tried to optimize parameters for versions 5.2 and 5.3 and searched for better parameters in the same ranges as done for version 5.1. With their respective default parameters, versions 5.1, 5.2, and 5.3 achieved on average accuracies of 0.23, 0.41, and 0.22 in 4.5, 3.5, and 2.2 min, respectively. During optimization, versions 5.2 and 5.3 were found to be less responsive to parameter tuning. The effects of changing each parameter while keeping others at default are shown in Supplemental Table S2 for version 5.2 and in Supplemental Table S3 for version 5.3. Most notably, changing ‘ne’ in version 5.2 had no effect. While ‘ne’ did affect the accuracies for version 5.3, optimizing parameters for this version was generally more difficult than for version 5.1. When run with the optimized parameters found for version 5.1 as presented in Table 3; 5.1, 5.2, and 5.3 achieved on average accuracies of 0.82, 0.40, and 0.72 in 209, 190, and 213 min, respectively.

## 4 DISCUSSION

### 4.1 Tuning parameters

Tuning parameters helped to improve accuracies, especially for Beagle which was developed for human data that typically show very different low‐density and homozygosity patterns than plants. Previous work by Pook et al. (2020) on maize DH lines showed that tuning Beagle's parameters can yield better imputation results. The parameters declared as optimal and different from default in this study deviate even more from default than those found by Pook et al. (2020). First, this might be caused by different population structures of the considered datasets. Second, the imputation procedure used in this study for parameter optimization differed from the one used by Pook et al. (2020). Third, a much larger range for parameters than in Pook et al. (2020) was considered. This is particularly relevant for those parameters controlling the number of iterations performed, as corner solutions (i.e., better than or equal to default values were at either border of the tested range) were reached in Pook et al. (2020), while we still observed improvement for a far higher number of iterations. Lastly, the number of markers and the number of lines used in this study was far lower, suggesting that the importance of optimization increases the less information is available. Note that the parameters described as ‘optimized’ in this study are specific to the sugar beet data we used. They were optimized for data sets with F3 offspring lines based on the real parent genotypes. Different parameters may be optimal for the type of data sets tested in our other scenarios, for example, for different population types especially those with more crossovers between parent and offspring lines. For better comparability, we did not optimize parameters for every scenario anew. For other data sets or crops, we recommend optimizing parameters for the intended use case.

Based on the observations presented in this study, we recommend using Beagle on plant breeding data only with adjusted parameters. For optimization of parameters in other data sets, we recommend to first change the value for the ‘window’ parameter so that the whole chromosome is covered if computing time allows. In a second step, the ‘ne’ parameter (effective population size) should be adjusted as this parameter has a large effect on accuracy. Figure 2a suggests that using a precisely calculated value of the effective population size does not matter as accuracies did not change for our data set for values in the range of 1 to 250. We suggest using a value of 100 for breeding populations when a precise estimate is not available. This value is supported by a number of publications which estimate effective population sizes for crop and livestock populations much closer to 100 than to 1 million which is the default setting (Cowling, 2007; Gorssen et al., 2020; Leroy et al., 2013; Makanjuola et al., 2020; Saura et al., 2021; Wilmot et al., 2020; Zhao et al., 2021). After the publication of Pook et al. (2020), some studies have already adopted lower values for ‘ne’ (Thorn et al., 2021; Whalen & Hickey, 2020), and a few are also increasing the window size (Arouisse et al., 2020; Geibel et al., 2021; Lamb et al., 2021).

The optimized value of 4 for ‘ne’ presented in this study was chosen after the optimization process during which multiple parameters were altered at a time. We do not expect that a different value, for example, 50, would have resulted in much different accuracies. Our proposed optimized choice is therefore somewhat arbitrary.

The parameter ‘iterations’ should be increased last because it significantly affects the run time. No negative effect of increasing the number of iterations was observed in this study. With respect to the linear increase in run time when increasing ‘iterations’ (Supplemental Figure S1), a potential approach to find a good value for ‘iterations’ may be to impute the data once with default settings (i.e., 12) and a second time with any other value. This allows to approximate the additional run time for one additional iteration which can then be used to calculate the maximum number of iterations that allow imputation within the time limit. Additional iterations do not guarantee better accuracies as found by Boison et al. (2015) for earlier versions of Beagle on cattle data.

Changing parameters did not affect the accuracies achieved with API2 as much as the ones achieved with Beagle. This does not surprise considering API2 was developed for plant breeding data. This is in line with reports of Thorn et al. (2021). After extensive search, we found parameters with which API2 performs slightly better. However, our findings do not object using default parameters which we would recommend for most practical applications.

### 4.2 Reflection on methods

Separating an imputation problem into two steps for phasing of high‐density genotypes followed by imputation of low‐density genotypes was found to be advantageous for Beagle (compare ‘1 step’ and ‘2 step’ for optimized parameters in plot A of Figure 1). Imputation with Beagle in two steps has been done in other studies before (Boison et al., 2015; Thorn et al., 2021). Many tools such as the here used AlphaPlantImpute2 have this separation implemented by default.

We initially used low‐density lines in the phasing step of the two‐step Beagle procedure as we believed that these lines may provide some additional useful information. However, we found that excluding them is advantageous (Supplemental Figure S2).

AlphaPlantImpute2 was found to be better in most scenarios in terms of accuracy. AlphaPlantImpute2 achieved this although it utilizes less information than Beagle as it does neither accept a genetic nor a physical map. However, its advantage over Beagle diminishes the more markers are used for genotyping (see effect of low‐density panels in Figure 3), the more lines are genotyped (see effect of number of high‐density offspring in Figure 3) and the less homozygous the genotypes are that have to be phased (compare population types based on F1 parents in Figure 5). This is in line with findings by Thorn et al. (2021). Its run time is considerably higher compared with default parameters in Beagle (Figure 1). The run time for the imputation step of API2 scales quadratically with the ‘n_haplotypes’ parameter (Thorn et al., 2021). In this study, the run time of API2 is mainly affected by the phasing step as our standard procedure of imputation considers pedigree information, thus reducing the number haplotypes sampled from the library to only those of the two parent lines.

Pedigree information improved imputation of API2 and Beagle slightly (for Beagle, compare ‘2 steps’ with ‘2 steps, imp per fam’ for Beagle in Figure 1a and see Supplemental Figure S3, for API2, compare ‘all for hap lib’ with ‘all for hap lib, imp w/ ped’ in Figure 1b). Pedigree information also reduced the run time for both software (see same comparisons in Figure 1c and Supplemental Figure S3 for Beagle, and Figure 1d for API2). Note that Beagle 5.1 is not able to use pedigree information on its own; manual work to adjust the reference panels is needed. Accounting for ancestor information is a new approach that is suggested in this manuscript. For Beagle, closer investigation showed that the restriction to the two respective parents only has a minor effect on accuracy while the main improvement of familywise imputation comes from the reduced number of genotypes in the imputation set (see Supplemental Figure S3) as discussed later. This suggests that Beagle is quite good in finding the right parents from the reference which has also been shown by Pook et al. (2020). We suspect that the effect of restricting the reference set may be higher when suboptimal parameter values are used.

Our findings suggest that Beagle is superior in phasing as shown by populations based on F1 parents in Figure 5. The F1 lines are the most heterozygous type tested and consequently the least phased by design thus representing the most difficult type to phase. We speculate that Beagle's superiority in phasing stems from its original purpose as an imputation tool for human genetics where heterozygous data is common. AlphaPlantImpute2 was developed for agricultural plant breeding where plant types with homozygosity levels much higher than those in F1s are typically of interest for genotyping. AlphaPlantImpute2 is superior in imputation as shown by populations with F1 offspring in Figure 5. The F1 offspring, especially when based on highly homozygous parent lines and known pedigree, are the easiest type to impute because they are the most related to the parents in terms of separating recombination events. In case F1 offspring is based on DH parents, the F1 is the average of the genotype states of the parents which allows the imputation accuracy to be 1. AlphaPlantImpute2 achieved this perfect accuracy, but Beagle did not (see boxplots for these population types in Figures 5a and 5b).

Note that we did not investigate phasing quality explicitly. Our interpretation of phasing performance is only based on the imputation accuracy of offspring lines. The phasing quality of parental lines is only indirectly of interest to improve imputation performance.

### 4.3 Reflection on new pipelines

We tested our new imputation pipelines ‘single‐genotype imputation in Beagle’ and ‘Beagle+API2’ in scenarios that have previously been found to be challenging for at least one software. The first scenario (Row 1 in Table 4) is our standard scenario used throughout this study. In the second scenario, a high number of low‐density offspring lines were to be imputed which has been found to be challenging for Beagle (see Figures 1, 7; Supplemental Figure S3; Table 4). This inspired us to design the single‐genotype imputation pipeline. The accuracy of ordinary Beagle imputation decreases with increasing number of low‐density lines because the genotype set provided for imputation affects the haplotype cluster and library in Beagle that initializes the Hidden Markov Model and thus the effective weighting of different founder haplotypes for the imputation pipeline. In other words, imputation is not only affected by the reference set but also by the set that is to be imputed. Although the lines in the imputation set may be closely related as it is the case in our familywise approach, sibling lines do not provide additional information if the parents are included in the reference as additional crossovers compared with the parents should only introduce noise.

The scenario in Row 3 of Table 4 (DH offspring based on F1 parents) has been found to be challenging for API2 as presented in Figure 5b. We interpreted that API2 is inferior in phasing compared with Beagle (discussed above). Scenario 4 (F1 offspring based on DH parents) was chosen because Beagle has previously been found to be inferior in imputation. This inspired us to design the Beagle+API2 pipeline which outperforms all other approaches or yields equal accuracies.

The last scenario was chosen because Beagle was found to be relatively worse in imputing low‐density parents based on high‐density offspring (Figure 6). This is the only scenario in which the single‐genotype imputation pipeline did not notably outperform the two‐step procedure in Beagle (see Table 4).

Coupling the strengths of both software in our Beagle+API2 pipeline resulted in the highest accuracies and fastest run time as shown in Table 4 and Supplemental Table S1, respectively. The properties of both software may be exploited for future development of imputation software development for populations with long‐range low‐density. Note that although we presented the Beagle+API2 approach without accounting for pedigree information, utilization of pedigree information can be integrated.

It should be noted that although Beagle was not the best tool in most of our tests, it is a general solution that can be applied to more different data sets than AlphaPlantImpute2 which was developed only for plant breeding.

The run times presented in this study should be interpreted as the relative advantage of one method over another. The elapsed wall clock time could be decreased further by running imputation in parallel per chromosome and per family or genotype. Single‐genotype imputation with Beagle would profit the most from parallelization. Much shorter run times per central processing unit can be achieved by using deterministic imputation software such as FImpute (Sargolzaei et al., 2014) or AlphaPlantImpute (Gonen et al., 2018).

### 4.4 Application

Low‐density genotyping of offspring may reduce genotyping costs. Together with resource saving, genotyping with a reduced marker set followed by imputation should be evaluated with regards to the effects imputed genomes have on the downstream analysis like genomic prediction or genome‐wide association studies. Moghaddar et al. (2015) report no negative effect on genomic prediction for imputation accuracies >0.95. Imputation of lines previously genotyped with a low‐density array can also make those lines usable in downstream applications and further increase the size of the training population.

However, imputing low‐density offspring is different from imputing progenitors. Offspring might show unique linkage at recombination points never observed before or in their sibling lines. Thus, imputing the regions around crossovers is particularly difficult. It can be mitigated by larger low‐density chips. Imputing low‐density progenitors has other implications. Imputation is relatively easier because the linkage pattern found in their genomes is largely passed on to the next generation genotyped at high‐density. Forces like selection and drift may cause the loss of alleles or haplotypes in the current population that were present in its progenitors’ genomes (Cowling, 2013). Such lost variation is impossible to accurately impute. Supplemental Figure S4 visualizes the two problems. We tested imputing low‐density offspring with high‐density parent data as a reference (Figure 3) and vice versa (Figure 6). While API2 always yielded higher accuracies in the latter scenario, the relative superiority of Beagle increased in the first scenario the more lines were genotyped at high‐density and the more markers were included on the low‐density array. This further supports the theory that the kind of relationship between reference and low‐density lines is an important factor to consider for the application.

For commercial breeding programs that routinely use newer and denser marker chips, this raises the question for the optimal strategy to do imputation over breeding cycles. A possible solution may be to impute chronologically backwards, that is, first the parents of the most recent generation are imputed. Then, the grandparents are imputed with the parents (grandparents’ offspring) as reference and so forth. This ensures that the haplotype segments in the reference and imputation set are as similar as possible. Boison et al. (2015) propose a similar sequential imputation strategy in which a subset of individuals with the highest genomic relationship to a fixed number of high‐density individuals is imputed first and then added to the reference set to impute other individuals. Moghaddar et al. (2015) improved the imputation accuracy by only including the 20 most related animals in the reference set for every low‐density animal in a study on Merino sheep. These two techniques may be extended to be applied on a chromosome level for further accuracy enhancement. Toghiani et al. (2016) investigate the imputation accuracy over several cycles when using a low‐density chip and the starting generation as a fixed high‐density reference. They substantially mitigated the imputation accuracy decay by updating the reference set with the high‐density genotypes of top animals every generation. This is in line with our findings that genotyping high‐density sibling lines in addition to parents increases the accuracy which is commonly reported in literature (Gonen et al., 2018; Hickey et al., 2012; Hickey et al., 2015; Whalen et al., 2020). If limited resources for genotyping are available, we thus propose genotyping the most promising lines if preliminary genomic breeding values are available. Alternatively, a fixed number of most related lines per parent can be genotyped starting from the most influential parent until genotyping resources are exhausted. This ensures that the reference set will be most relevant for most lines.

### 4.5 Missing and incorrect genotype calls

The effect of various proportions of missing and incorrect genotype calls on imputation accuracy was investigated. AlphaPlantImpute2 performed slightly better than Beagle in the presence of missing genotype calls, whereas Beagle outperformed API2 when incorrect genotype calls were induced (see Figure 4a, 4b). In practice, missing calls and genotyping errors are common (Beissinger et al., 2013; Unterseer et al., 2014), but their occurrence rates are affected by the genotyping technique and population diversity. The chemistry of the SNP arrays can also influence error rates as described by Wiggans et al. (2012). The exact properties for the experimental chip simulated by us are unknown making it difficult to translate the presented findings in a general suggestion.

The sensitivity of the software to missingness and genotyping errors might change the more lines and markers are genotyped. Functional reasons like deletions, may cause missing calls which would result in some sites being more affected than others. Different alleles in segmental duplications, which are retained in the ancient polyploid maize genome for example, may appear as heterozygous calls in homozygous lines (Schnable et al., 2009; Unterseer et al., 2014) and might be deemed genotyping errors. Note that we modelled random errors and missingness as done in other studies (Forneris et al., 2015; Pook et al., 2020) which thus does not reflect how real data sets are affected.

A strategy to deal with problematic marker can be to use the R2 estimate provided by Beagle. Such poorly predicted markers could then be excluded from the data set prior to downstream applications as has been suggested by Boison et al. (2015) and Pook et al. (2020). An alternative general strategy to deal with problematic markers is to exclude markers with high percentages of missingness, parent‐offspring conflicts and Hardy–Weinberg equilibrium discrepancies prior to imputation as suggested by Wiggans et al. (2012) for dairy cattle. Minor allele frequency filtering may also be applied for quality control but poses the risk of removing true rare variants. Forneris et al. (2015) propose a method to detect loci for which a large number of animals have incorrect genotype states by considering allele count as a quantitative trait and testing whether heritability deviates from 1.

### 4.6 Population types

The findings of other studies stating a positive effect of the homozygosity level of parents on imputation accuracy could be confirmed (Gonen et al., 2018; Hickey et al., 2015). Those parts of the genome that are homozygous are effectively phased. Thus, the phasing step is not necessary for fully inbred parents as they are phased by design (Pook et al., 2021).

A negative trend between imputation accuracy and the number of meiotic events separating parents from offspring generation has been found for open‐pollinating populations and selfing populations. The more generations are separating parents from offspring, the more crossovers can break down linkage between alleles as seen in the parents. As an example, double haploid and F10 lines can both be considered fully inbred. Thus, if the homozygosity level was affecting the imputation accuracy, no difference in accuracy between F10 and DH offspring should be found. As shown in Figure 5a and 5b, the contrary is the case with DH offspring lines resulting in higher accuracies than F10 offspring lines within any parent type regardless of the software. This can be attributed to the fact that F10 lines show twice as many effective crossovers than DH lines. As explained above and shown in Supplemental Figure S4, imputing alleles around recombination points is imprecise.

The comparison of imputation accuracy for different population types is informative for designing imputation tools or from a strategic perspective, for example, in decisions about which density to use on the array. For direct breeding applications, it may be less useful to know which population type results in the highest accuracy because breeding programs will not change the type of lines for the purpose of increasing accuracy. The comparison of different population types as shown in Figures 5 and 6 and in Table 1 is useful to decide which software to use for a given breeding program.

### 4.7 Beagle versions

Beagle 5.2 performed better than versions 5.1 and 5.3 when using default parameters and setting parameter ‘em’ to ‘false’ in Beagle 5.3. Changing parameter ‘ne’ had an effect for versions 5.1 and 5.3 but not for version 5.2. This is because Beagle 5.2 estimates a value for ‘ne’ based on the data in the window (Browning et al., 2021) only using the specified value as a starting point for estimation. In our dataset, Beagle 5.2 estimated values in the high hundreds. Based on observations with Beagle 5.1 (Figure 2a), the best values for ‘ne’ are however below 250. Thus, we believed that the estimation of the effective population size may not be accurate for plant breeding data. We communicated this finding with the developer Brian Browning who then released version 5.3 in which the automated estimation of the effective population size can be turned off by setting ‘em’ to ‘false’. Although accuracies of up to 0.72 were achieved with 5.3, the best imputation quality among all Beagle versions was realized with 5.1. The ranking of the versions may depend on the population structure and marker density. It should be noted that versions 5.2 and 5.3 are developed to run much faster which will be advantageous for large datasets.

## 5 CONCLUSIONS

Our findings inform breeders seeking to obtain full high‐quality data even when only a small fraction of markers is genotyped. Thus, imputation can be used to reduce costs in plant breeding. Though the results presented in this paper are based on sugar beet, the findings should be transferable to other crops as the genetic architecture of datasets should be comparable. For more fair comparisons, future work in plant breeding imputation should not use imputation software with default settings that were developed for other purposes. Our tests suggest that API2 is a powerful tool for the imputation of plant breeding data and in particular datasets with limited genetic diversity or pedigree information. Beagle 5.1 led to competitive results when adapting parameter settings as well as modifying the imputation procedure and should be more versatile on data panels with more diversity than the panel considered here. The suggested pipeline herein (Beagle+API2), combining the strengths of both tools, led to the highest accuracies in the shortest run times. Default settings of Beagle were shown to be not suitable for the here considered datasets. Among the Beagle versions, Beagle 5.1 could be optimized the most resulting in higher accuracies than 5.2 and 5.3.

## AUTHOR CONTRIBUTIONS

Tobias Niehoff: Conceptualization; Formal analysis; Investigation; Methodology; Validation; Visualization; Writing – original draft; Writing – review & editing. Torsten Pook: Conceptualization; Methodology; Supervision; Writing – review & editing. Mahmood Gholami: Conceptualization; Data curation; Supervision; Writing – review & editing. Timothy Beissinger: Conceptualization; Project administration; Supervision; Writing – review & editing.

## CONFLICT OF INTEREST

The authors declare that they have no competing interests.

## Supporting information

Supplementary Figure S1: Effect of the number of iterations on imputation accuracy (A) and run time (B) of Beagle. For all other parameters other than ‘iterations’, the optimized values presented in Table 3 were used. The black line indicates the default value for iterations (12). The imputation procedure used Beagle in two steps without utilizing pedigree information (noted as ‘2 steps’ in plot A, Figure 1).
Supplementary Figure S2: Effect of including low‐density offspring in phasing/haplotype library creation step of Beagle and AlphaPlantImpute2, respectively, for different population types. (A) refers to imputation with Beagle; (B) refers to imputation with API2. Note that the standard procedure of Beagle and API2 differ with regards to whether low‐density individuals are used for phasing. In all cases, low‐density lines are removed from the reference set/library prior to imputation.
Figure S3: Effect of reducing number of individuals in genotype set for imputation and utilization of parent information by Beagle. The leftmost procedure is the one used as standard in this paper (familywise imputation). The second from left procedure is identical except that not only the two parents of a cross but all 30 parents are provided in the reference. The procedure on the right does not use familywise imputation (2 step procedure).
Supplementary Figure S4: Offspring vs. parent imputation (schematic). The species in this example is haploid. Alleles from parent 1 (P1) shown in orange and blue for parent 2. O1 and O2 are offspring of the cross P1 x P2. Imputation in this example fills the space between two alleles inherited from the same parent with the alleles from that parent. Loci surrounded by allelels from different parents cannot be imputed as the crosssover can be anywhere in between.
Supplementary Table S1: Run times of different imputation methods for selected scenarios. No pedigree information was used. The run time is reported in minutes. The standard error of the mean is reported in parentheses.
Supplementary Table S2: Ranges of accuracies when varying parameters in Beagle 5.2 and using the 2‐step procedure. The ranges of the best parameters are indicated if this was supported by a clear trend. Within the ranges indicated in the column labelled ‘best’, accuracy varied by about 0.02. Hyphens indicate that no trend supported stating a value as best. Variation in accuracy was thus attributed to random fluctuation.
Supplementary Table S3: Ranges of accuracies when varying parameters in Beagle 5.3 and using the 2‐step procedure with *em=false*. The ranges of the best parameters are indicated if this was supported by a clear trend. Within the ranges indicated in the column labelled ‘best’, accuracy varied by about 0.02. Hyphens indicate that no trend supported stating a value as best. Variation in accuracy was thus attributed to random fluctuation.

## Data Availability

The genotype data and genetic map are proprietary to KWS SAAT SE & Co. KGaA. Data of the first chromosome can be obtained from the authors upon reasonable request. The R scripts for the imputation procedures are available in the GitHub repository. For testing purposes, we also provide a simple dummy dataset.
