# Supplementary material for: Imputation of low‐density marker chip data in plant breeding: Evaluation of methods based on sugar beet
Source: Plant Genome. 2022 Oct 18;15(4):e20257. doi: 10.1002/tpg2.20257 (PMC12807303; doi:10.1002/tpg2.20257)
Supplement: Supplementary file 1 [file TPG2-15-e20257-s001.zip › SupplementaryFigure S1.docx]

**A**

**B**

Supplementary Figure S1: Effect of the number of iterations on imputation accuracy (A) and run time (B) of Beagle. For all other parameters other than ‘iterations’, the optimized values presented in Table 3 were used. The black line indicates the default value for iterations (12). The imputation procedure used Beagle in two steps without utilizing pedigree information (noted as ‘2 steps’ in plot A, Figure 1).
