# Supplementary material for: Imputation of low‐density marker chip data in plant breeding: Evaluation of methods based on sugar beet
Source: Plant Genome. 2022 Oct 18;15(4):e20257. doi: 10.1002/tpg2.20257 (PMC12807303; doi:10.1002/tpg2.20257)
Supplement: Supplementary file 1 [file TPG2-15-e20257-s001.zip › SupplementaryFigure S2.docx]

**A**

**B**

Supplementary Figure S2: Effect of including low-density offspring in phasing/haplotype library creation step of Beagle and AlphaPlantImpute2, respectively, for different population types. (A) refers to imputation with Beagle; (B) refers to imputation with API2. Note that the standard procedure of Beagle and API2 differ with regards to whether low-density individuals are used for phasing. In all cases, low-density lines are removed from the reference set/library prior to imputation.
