# Supplementary material for: Imputation of low‐density marker chip data in plant breeding: Evaluation of methods based on sugar beet
Source: Plant Genome. 2022 Oct 18;15(4):e20257. doi: 10.1002/tpg2.20257 (PMC12807303; doi:10.1002/tpg2.20257)
Supplement: Supplementary file 1 [file TPG2-15-e20257-s001.zip › SupplementaryFigure S4.docx]

| (A) Imputation of offspring |  |  |
| --- | --- | --- |
|  | P1 (HD) |  |
|  | P2 (HD) |  |
|  | O1 (LD) |  |
|  | O1 (imputed only with known haplotypes) |  |
|  | O1 (true) |  |
| (B) Imputation of parent |  |  |
|  | O1 (HD) |  |
|  | O2 (HD) |  |
|  | P1 (LD) |  |
|  | P1 (imputed only with known haplotypes) |  |
|  | P1 (true) |  |

Supplementary Figure S4: Offspring vs. parent imputation (schematic). The species in this example is haploid. Alleles from parent 1 (P1) shown in orange and blue for parent 2. O1 and O2 are offspring of the cross P1 x P2. Imputation in this example fills the space between two alleles inherited from the same parent with the alleles from that parent. Loci surrounded by allelels from different parents cannot be imputed as the crosssover can be anywhere in between.
